# Longitudinal data on implementing an activity-based work environment

**DOI:** 10.1016/j.dib.2022.107920

**Published:** 2022-02-04

**Authors:** Freyr Halldorsson, Kari Kristinsson, Svala Gudmundsdottir, Lilja Hardardottir

**Affiliations:** a Reykjavik University, Menntavegur 1, Reykjavik 102, Iceland; b University of Iceland, Sæmundagata 2, Reykjavik 102, Iceland

**Keywords:** Activity-based working, Employee attitudes, Longitudinal, Employee outcomes

## Abstract

Using a longitudinal field survey, we collected data on how implementing an activity-based work environment impacts employees across time [1]. The sample consisted of 100 employees in a government organization implementing an activity-based working environment, with each employee surveyed on three time-points. The sample included all employees affected by the implementation. At each time-point, the response rate was 87%, 75%, and 69%, respectively. The sample was approximately 75% female at each time-point. Data collection took place about two months before the activity-based environment was implemented (condition 1), again about four months after implementation (condition 2), and finally, about nine months after implementation (condition 3). All data were collected using an online survey. The survey included questions on privacy, psychological ownership, and attitude towards activity-based work, in addition to questions on productivity, job satisfaction, job strain, and satisfaction with the work environment.

## Specifications Table

**Table:** 

Subject	Applied Psychology.
Specific subject area	The effects of implementing an activity-based work environment.
Type of data	Table (Excel).
How data were acquired	An online survey was administered via www.QuestionPro.com. The survey is provided as supplementary material.
Data format	Raw (see http://dx.doi.org/10.17632/w359fz96rb.4).
Parameters for data collection	Employees experienced a move from a traditional office space—a mix of private offices and assigned cubicles in an open plan. The new work environment was fully activity-based, with no offices and no assigned workstations. The sample included all affected employees.
Description of data collection	Data were collected with an online survey at three time-points during the implementation of an activity-based work environment. Data collection took place about two months before the activity-based environment was implemented (condition 1), again about four months after implementation (condition 2), and finally, about nine months after implementation (condition 3). Each time, employees received a link via email, inviting them to participate, followed by two reminders.
Data source location	Institution: Reykjavik University City/Town/Region: Reykjavik Country: Iceland Latitude and longitude: 64.123753, -21.926866
Data accessibility	Halldorsson, Freyr; Kristinsson, Kari; Gudmundsdottir, Svala; Hardardottir, Lilja (2021), “Longitudinal Data on Implementing an Activity-Based Work Environment”, Mendeley Data, v4, DOI: 10.17632/w359fz96rb.4 Repository name: Mendeley Data Data identification number: 10.17632/w359fz96rb.4 Direct URL to data: http://dx.doi.org/10.17632/w359fz96rb.4
Related research article	Halldorsson, F., Kristinsson, K., Gudmundsdottir, S., & Hardardottir, L. (2021). Implementing an Activity-Based Work Environment: A Longitudinal View on the Role of Privacy and Psychological Ownership, *Journal of Environmental Psychology, 78*, 101,707. 10.1016/j.jenvp.2021.101707

## Value of the Data

• This data provides insight into how implementing activity-based work environments may impact employees over time. The data includes measures of several psychological constructs, including privacy, psychological ownership, perceived productivity, job satisfaction, and job strain.
• This data can be helpful to researchers interested in the effects of work environments on employees, especially activity-based work environments. The data may also be beneficial to managers of organizations that are considering implementing activity-based work environments.
• The data could be reanalyzed using more sophisticated statistical tools and provide researchers with the possibility of testing novel hypotheses.

## 1 Data Description

The data includes responses from a sample of 100 employees working at a government organization that was implementing an activity-based working environment. The sample included all employees affected by the implementation. Each participant was surveyed three times with response rates of 87%, 75%, and 69%, respectively. The sample was approximately 75% female at each measurement point. By comparison, the organization was actually about 72% female at the time, so the sample is fairly representative with regards to gender. To maintain participant anonymity—given the relatively small size of the organization—only a single background variable was included, i.e., gender. Based on the organization's annual report, the average age of employees was about 50 years and 48% had a university degree (bachelor's degree or higher).

A Word file (``Survey—Longitudinal Data on Implementing Activity-Based Work Environment.docx'') includes all administered survey items. The four questions that are reverse worded are indicated with an [R]. See Table 1 for a list of all measures included in the survey and a brief description.

**Table 1:** Survey measures and descriptions.

Measure	Description
*Productivity*	Three items were adapted from Rolfö et al. [2]. Uses a 7-point Likert scale ranging from 'strongly disagree' to 'strongly agree.'
*Privacy*	Privacy was measured using six items from Oldham [3]. The original measure captures two dimensions—'task privacy' and 'communication privacy’—but all items were combined into a single score, following Laurence et al. [4]. The construct was captured using a 7-point Likert scale ranging from 'Strongly disagree' to 'Strongly agree.'
*Psychological ownership*	Four items from Brown [5]. The scale was captured using a 7-point Likert scale ranging from 'Strongly disagree' to 'Strongly agree.'
*Job satisfaction*	Two items from Veitch et al. [6] using a 7-point Likert scale ranging from 'Strongly disagree' to 'Strongly agree.'
*Attitude towards activity-based work environments (ABW-attitude)*	Single item: "Overall, I have a positive attitude towards an activity-based work environment," created for the study and intended to capture participants' overall attitude towards activity-based work. Participants responded to the item using a 7-point Likert scale—ranging from 'Strongly disagree' to 'Strongly agree.'
*Workload*	Two items captured workload using a 7-point Likert scale ranging from 'Strongly disagree' to 'Strongly agree.'
*Job strain*	Four items captured job strain using a 4-point scale ((1) Almost never, (2) Rarely, (3) Sometimes, (4) Almost daily).
*Work environment satisfaction*	Participants were asked to rate how satisfied they were with their work environment using a 7-point Likert scale ranging from 'Very unsatisfactory' to 'Very satisfactory.' The measure includes 18 specific factors (e.g., air quality, lighting, temperature, etc.) and an overall rating of the work environment.
*How often do you normally switch workplaces?*	A single item that captures how often employees choose a different place to work within the activity-based work environment, ranging from "Never" to "Often per day."
*Effects on performance*	Participants were asked to estimate the effects of the work environment on their performance in recent days.
*Gender*	The gender of respondents (1= female, 2=male)

Descriptive statistics for the scales across each measurement point can be seen in Table 2, including, number of observations (n), minimum and maximum values, means, and standard deviations.

**Table 2:** Descriptive statistics for scales across measurement points.

Variable	n	Min.	Max.	Mean	St.dev.
Productivity—T1	79	2.67	7.00	5.85	.92
Productivity—T2	70	3.00	7.00	5.61	1.07
Productivity—T3	61	2.00	7.00	5.57	1.07

Privacy—T1	79	2.00	7.00	4.89	1.19
Privacy—T2	69	1.50	7.00	4.48	1.37
Privacy—T3	60	2.00	6.83	4.34	1.39

Psychological ownership—T1	80	2.00	7.00	5.75	1.31
Psychological ownership —T2	67	1.00	7.00	3.66	1.90
Psychological ownership —T3	60	1.00	7.00	3.88	1.87

Job satisfaction—T1	79	2.00	7.00	5.35	1.31
Job satisfaction —T2	69	1.50	7.00	5.20	1.38
Job satisfaction —T3	60	1.50	7.00	5.33	1.39

ABW-attitude—T1	80	1.00	7.00	5.21	1.72
ABW-attitude —T2	54	1.00	7.00	5.24	1.73
ABW-attitude —T3	57	1.00	7.00	5.32	1.83

Workload—T1	79	1.00	7.00	3.23	1.48
Workload —T2	74	1.00	7.00	3.81	1.44
Workload —T3	69	1.00	7.00	3.28	1.41

Job strain—T1	80	1.00	5.00	2.30	.83
Job strain —T2	66	1.00	4.00	2.22	.86
Job strain —T3	59	1.00	4.50	2.34	.93

Work environm. satisfaction—T1	80	1.00	6.94	4.77	1.38
Work environm. satisfaction —T2	72	2.39	7.00	4.70	1.30
Work environm. satisfaction —T3	67	1.72	7.00	4.81	1.19

An Excel file (Data—Longitudinal Data on Implementing Activity-Based Work Environment.xlsx") contains the raw data for all variables collected across the three measurement points.

## 2 Experimental Design, Materials and Methods

The data were collected using longitudinal field design, measuring employee attitudes once before an activity-based environment was implemented and twice after implementation. Specifically, the data collection took place about two months before the activity-based environment was implemented (condition 1), again about four months after implementation (condition 2), and finally, about nine months after implementation (condition 3). The physical work environment in conditions 2 and 3 was the same—the only difference being that employees had spent more time in the new work environment in condition 3.

The online survey software QuestionPro (www.questionpro.com) was used to create an online survey that included all survey items. The same software was used to send the survey to potential participants using a list of email addresses obtained from the organization in question. For the initial survey (condition 1), an email was sent to all 100 potential participants with a brief description of the study and a link to open the survey. Before responding to survey items, all participants needed to provide consent for their participation in the study. As part of the consent process, it was explicitly stated that participation was voluntary and that participants could withdraw at any time without any consequences. It was also noted that participants could skip any items on the surveys if they did not want to respond. Subsequent surveys (for conditions 2 and 3) were only sent to those who consented to participate in the study. An email with a link to the survey was sent to participants for each data collection period, followed by two reminders.

Once the final data collection phase was completed, all data was downloaded and merged into a single Excel spreadsheet. The data was then cleaned by removing all columns with non-relevant or potentially identifiable information (e.g., time stamps, IP addresses, geolocation, etc.). The final data set included only an anonymized response ID and participants' responses to the survey items for each measurement period. Note that since participants were surveyed repeatedly, the structure of the data is hierarchical in nature with multiple measurements (within-person level) nested in each participant (between-person level).

## Ethics Statement

The authors declare that they comply with all the guidelines given by the Science Ethics Committee at the University of Iceland. Informed consent was obtained from all the participants, and all the data were anonymized.

## CRediT authorship contribution statement

**Freyr Halldorsson:** Supervision, Conceptualization, Methodology, Data curation, Writing – original draft, Writing – review & editing. **Kari Kristinsson:** Supervision, Methodology, Writing – review & editing. **Svala Gudmundsdottir:** Supervision, Visualization, Investigation. **Lilja Hardardottir:** Conceptualization, Investigation, Software.

## Declaration of Competing Interest

This research did not receive any specific grant from funding agencies in the public, commercial, or not-for-profit sectors. The authors declare that they have no known competing financial interests or personal relationships which have or could be perceived to have influenced the work reported in this article.

## Appendix Supplementary materials

Image, application 1

Image, application 2
