# Supplementary material for: Longitudinal data on implementing an activity-based work environment
Source: Data Brief. 2022 Feb 4;41:107920. doi: 10.1016/j.dib.2022.107920 (PMC8844190; doi:10.1016/j.dib.2022.107920)
Supplement: Supplementary file 1 [file mmc1.docx]

**Survey Items**

In this first part, you are asked to assess the following factors on a 7-point scale ranging from 'Strongly disagree' to 'Strongly agree'.

Productivity

- I feel that I work efficiently
- I feel that I work efficiently when I collaborate with my co-workers
- I feel that I am highly productive at my workstation

Workload

- The workload in my job is reasonable
- The workload in my job has increased in recent months [R]

Privacy

- I am able to concentrate fully on my while at work
- While at my workstation, I can work with few distractions or interruptions
- Interruptions at work often prevent me from giving my full attention to my job [R]
- I can talk to my co-workers in confidence while at my workstation
- It's difficult to work at my station because I have to worry about disturbing others' [R]
- I am unable to have personal or private discussions while at work [R]

Job satisfaction [JobSat]

- My department/agency is a good place to work
- I am satisfied with my job

Attitude towards activity-based work environments [ABW]

- Overall, I have a positive attitude towards an activity-based work environment

The following questions about your workspace, refer to your day-to-day work environment. Responses are on a 7-point scale from 'Strongly disagree' to 'Strongly agree'.

Psychological ownership [PsyOwn]

- This is my workspace
- I sense that this workspace is mine
- I feel a very high degree of personal ownership for this workspace
- I sense that this is my workspace

Work environment satisfaction [WorkE]

Please note that you are asked to assess the following factors on a 7-point scale from 'Very unsatisfactory' to 'Very satisfactory'.

- The amount of noise from other people's conversations when you're at your workstation
- Frequency of disruptions from other people
- Degree of enclosure of your work area by walls, screens or furniture
- Level of visual privacy within your office
- Distance between you and other people you work with
- Level of privacy for conversations in your office
- Amount of background noise (i.e., not speech) you hear at your workstation
- Size of your personal workspace to accommodate your work, materials, and visitors
- Your ability to alter physical conditions in your work area
- Aesthetic appearance of your office
- Air movement in your work area
- Overall air quality in your work area
- Temperature in your work area
- Quality of lighting in your works area
- Amount of lighting on the desktop
- Amount of light for computer work
- Amount of reflected light or glare in the computer screen
- Your access to a view of outside from where you sit
- My overall work environment

Self-assessed performance [Performance]
In the following question, you are asked to assess how much positive or negative impact your work environment has had on your performance over the past few days.

- The effect of the work environment on my performance
  - Lower performance 30% 20% 10% no effect 10% 20% 30% Higher performance

Job strain

The following questions are answered on a 4-point scale: 1) Almost never, 2) Rarely, 3) Sometimes, 4) Almost daily

- Does it occur frequently or rarely after the workday ends that…
  - you find it hard to stop thinking about work so that it negatively affects your spare time or personal life?
  - you're worried that an issue will arise at work that you can't solve?
  - you don't feel like going to work the next day?
  - You're so tired that you have trouble getting yourself to do anything?

How often do you normally switch workplaces? [Switch]

- - Never
  - Less than 1 time per week
  - 1–2 times a week
  - 3–4 times a week
  - At the beginning of each day
  - Once a day
  - Often per day

Gender

- - Female
  - Male
